# [2-(2,3-Dimethyl­anilino)phen­yl]methanol

**DOI:** 10.1107/S1600536810033325

**Published:** 2010-08-21

**Authors:** Nasirullah, Nazar Ul Islam, M. Nawaz Tahir, Ikhtiar Khan

**Affiliations:** a Institute of Chemical Sciences, University of Peshawar, Peshawar, Pakistan; b Department of Physics, University of Sargodha, Sargodha, Pakistan

## Abstract

In the title compound, C_15_H_17_NO, the 2,3-dimethyl­phenyl group is disordered over two sites with an occupancy ratio of 0.869 (3):0.131 (3). The major and minor components of the 2,3-dimethyl­anilino group are planar, with r.m.s. deviations of 0.0214 and 0.0303 Å, respectively, and are oriented at a dihedral angle of 2.6 (6)°. The phenyl­methanol–benzene ring is oriented at dihedral angles of 83.16 (6) and 81.0 (3)° with respect to the major and minor components of the 2,3-dimethyl­anilino group, respectively. An *S*(6) ring motif is present due to intra­molecular N—H⋯O hydrogen bonding. In the crystal, mol­ecules are connected into supra­molecular chains *via* O—H⋯O hydrogen bonding along the *b* axis. C—H⋯π inter­actions help to stabilize the crystal structure.

## Related literature

For a related structure, see: Nawaz *et al.* (2007 ▶). For graph-set notation, see: Bernstein *et al.* (1995 ▶).
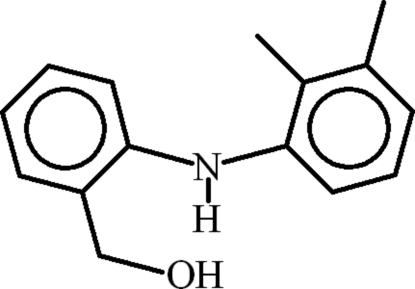


## Experimental

### 

#### Crystal data

C_15_H_17_NO
*M* *_r_* = 227.30
Monoclinic, 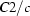

*a* = 26.819 (2) Å
*b* = 5.0317 (4) Å
*c* = 21.4156 (15) Å
β = 118.198 (3)°
*V* = 2547.0 (3) Å^3^
*Z* = 8
Mo *K*α radiation
μ = 0.07 mm^−1^
*T* = 296 K
0.34 × 0.25 × 0.22 mm

#### Data collection

Bruker Kappa APEXII CCD diffractometer
Absorption correction: multi-scan (*SADABS*; Bruker, 2005 ▶) *T*_min_ = 0.966, *T*_max_ = 0.975
9889 measured reflections
2298 independent reflections
1342 reflections with *I* > 2σ(*I*)
*R*_int_ = 0.051

#### Refinement

*R*[*F* ^2^ > 2σ(*F* ^2^)] = 0.052
*wR*(*F* ^2^) = 0.150
*S* = 1.03
2298 reflections
160 parameters
3 restraints
H-atom parameters constrained
Δρ_max_ = 0.21 e Å^−3^
Δρ_min_ = −0.16 e Å^−3^

### 

Data collection: *APEX2* (Bruker, 2009 ▶); cell refinement: *SAINT* (Bruker, 2009 ▶); data reduction: *SAINT*; program(s) used to solve structure: *SHELXS97* (Sheldrick, 2008 ▶); program(s) used to refine structure: *SHELXL97* (Sheldrick, 2008 ▶); molecular graphics: *ORTEP-3 for Windows* (Farrugia, 1997 ▶) and *PLATON* (Spek, 2009 ▶); software used to prepare material for publication: *WinGX* (Farrugia, 1999 ▶) and *PLATON*.

## Supplementary Material

Crystal structure: contains datablocks global, I. DOI: 10.1107/S1600536810033325/tk2701sup1.cif

Structure factors: contains datablocks I. DOI: 10.1107/S1600536810033325/tk2701Isup2.hkl

Additional supplementary materials:  crystallographic information; 3D view; checkCIF report

## Figures and Tables

**Table 1:** Hydrogen-bond geometry (Å, °) *Cg*1 and *Cg*2 are the centroids of the C1*A*—C6*A* and C9—C14 rings, respectively.

*D*—H⋯*A*	*D*—H	H⋯*A*	*D*⋯*A*	*D*—H⋯*A*
N1—H1⋯O1	0.86	2.35	2.904 (3)	123
O1—H1*A*⋯O1^i^	0.82	2.00	2.796 (2)	163
C8*A*—H8*A*⋯*Cg*1^ii^	0.96	2.88	3.783 (4)	157
C15—H15*B*⋯*Cg*2^iii^	0.97	2.77	3.634 (3)	148

Symmetry codes: (i) 
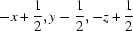
; (ii) 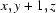; (iii) 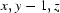.

**Fig. 1.**
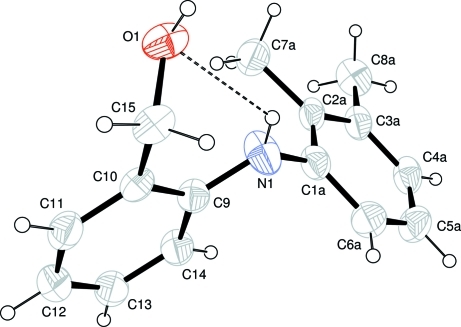
View of (I) with the atom numbering scheme showing atoms of the major component of the disorder. The displacement ellipsoids are drawn at the 50% probability level. H-atoms are shown as small spheres of arbitrary radii. The dotted line represent the intramolecular H-bonding.

**Fig. 2.**
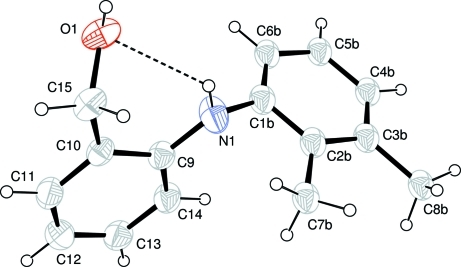
View of (I) with the atom numbering scheme showing atoms of the minor component of the disorder. The displacement ellipsoids are drawn at the 50% probability level. H-atoms are shown as small spheres of arbitrary radii. The dotted line represent the intramolecular H-bonding.

**Fig. 3.**
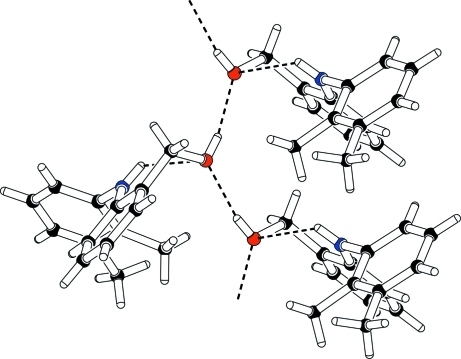
The partial packing (PLATON; Spek, 2009) showing that molecules are interlinked and form supramolecular chains via O–H···O hydrogen bonding (dashed lines). The intramolecular S(6) ring motifs are also illustrated (dashed lines). Only the major component of the disordered group is shown for clarity.

## supplementary crystallographic information

### Comment

The title compound (I, Fig. 1) is an important intermediate in our research related to the synthesis of pharmaceutically important derivatives of commonly used drugs. In this context, the crystal structure of a compound related to (I), *i.e*. methyl 2-(2,3-dimethylanilino)benzoate (Nawaz *et al.*, 2007), has been published recently.

In (I), the 2,3-dimethylphenyl group is disordered over two sites with an occupancy ratio of 0.869 (3):0.131 (3). The (2,3-dimethylphenyl)amino groups A (C1A—C8A/N1) and B (C1B—C8B/N1) are each planar with r. m. s. deviation of 0.0214 and 0.0303 Å, respectively. The dihedral angle between A/B is 2.4 (6) °. The dihedral angles between A/C and B/C are 83.16 (6) and 81.0 (3) °, respectively; C is the least-square plane through the benzene ring. An S(6) ring motif (Bernstein *et al.*, 1995) is formed due to intramolecular H-bond of the type N—H···O (Fig. 1). The molecules associate into supramolecular chains (Fig. 2) via H-bonding of the type O—H···O along the *b* axis. The presence of C—H···π interactions (Table 1) also play an important role in stabilizing the crystal structure.

### Experimental

A solution of mefenamic acid (8.2 mmol) in THF (20 ml) was slowly added to a suspension of NaBH_4_ (10 mmol) in THF (20 ml), at room temperature. The mixture was stirred until evolution of hydrogen ceased. Iodine (4.1 mmol) in THF (20 ml) was added drop wise to this mixture. When the addition of iodine was complete, the reaction mixture was refluxed for 8 h and cooled to room temperature. Then, 2 N HCl (10 ml) was added and the mixture was extracted with ether. The ether layer was washed with 2 N NaOH (20 ml) and then with brine. Finally, the ether layer was dried over MgSO_4_. Evaporation of solvent yielded a mixture of mefenamic acid and an alcohol. The pure product was obtained by passing the mixture over silica gel column (eluent: *n*-hexane and ethyl acetate). The product, (I), was recrystallized from ethyl acetate and *n*-hexane. The yield of reaction was 73%; m.pt. 337 K.

### Refinement

The H atoms were positioned geometrically (O—H = 0.82, N—H = 0.86 and C–H = 0.93–0.97 Å) and refined as riding with *U*_iso_(H) = x*U*_eq_(C, N, O), where x = 1.5 for methyl H atoms and x = 1.2 for all other H atoms. The 2,3-dimethylphenyl ring was found to be disordered and was resolved over two positions with an occupancy ratio of 0.869 (3):0.131 (3). Each ring was treated as a regular hexagon and the anisotropic displacement parameters for equivalent atoms were constrained to be equivalent.

### Crystal data

**Table:**

C_15_H_17_NO	*F*(000) = 976
*M**_r_* = 227.30	*D*_x_ = 1.186 Mg m^−^^3^
Monoclinic, *C*2/*c*	Mo *K*α radiation, λ = 0.71073 Å
Hall symbol: -C 2yc	Cell parameters from 1342 reflections
*a* = 26.819 (2) Å	θ = 3.1–25.2°
*b* = 5.0317 (4) Å	µ = 0.07 mm^−^^1^
*c* = 21.4156 (15) Å	*T* = 296 K
β = 118.198 (3)°	Prism, dark-red
*V* = 2547.0 (3) Å^3^	0.34 × 0.25 × 0.22 mm
*Z* = 8	

### Data collection

**Table:**

Bruker Kappa APEXII CCD diffractometer	2298 independent reflections
Radiation source: fine-focus sealed tube	1342 reflections with *I* > 2σ(*I*)
graphite	*R*_int_ = 0.051
Detector resolution: 8.10 pixels mm^-1^	θ_max_ = 25.2°, θ_min_ = 3.1°
ω scans	*h* = −32→32
Absorption correction: multi-scan (*SADABS*; Bruker, 2005)	*k* = −6→4
*T*_min_ = 0.966, *T*_max_ = 0.975	*l* = −25→25
9889 measured reflections	

### Refinement

**Table:**

Refinement on *F*^2^	Primary atom site location: structure-invariant direct methods
Least-squares matrix: full	Secondary atom site location: difference Fourier map
*R*[*F*^2^ > 2σ(*F*^2^)] = 0.052	Hydrogen site location: inferred from neighbouring sites
*wR*(*F*^2^) = 0.150	H-atom parameters constrained
*S* = 1.03	*w* = 1/[σ^2^(*F*_o_^2^) + (0.0649*P*)^2^ + 0.7177*P*] where *P* = (*F*_o_^2^ + 2*F*_c_^2^)/3
2298 reflections	(Δ/σ)_max_ < 0.001
160 parameters	Δρ_max_ = 0.21 e Å^−^^3^
3 restraints	Δρ_min_ = −0.16 e Å^−^^3^

### Special details

**Table:**

Geometry. Bond distances, angles *etc*. have been calculated using the rounded fractional coordinates. All su's are estimated from the variances of the (full) variance-covariance matrix. The cell e.s.d.'s are taken into account in the estimation of distances, angles and torsion angles
Refinement. Refinement of *F*^2^ against ALL reflections. The weighted *R*-factor *wR* and goodness of fit *S* are based on *F*^2^, conventional *R*-factors *R* are based on *F*, with *F* set to zero for negative *F*^2^. The threshold expression of *F*^2^ > σ(*F*^2^) is used only for calculating *R*-factors(gt) *etc*. and is not relevant to the choice of reflections for refinement. *R*-factors based on *F*^2^ are statistically about twice as large as those based on *F*, and *R*- factors based on ALL data will be even larger.

### Fractional atomic coordinates and isotropic or equivalent isotropic displacement parameters (Å^2^)

**Table:**

	*x*	*y*	*z*	*U*_iso_*/*U*_eq_	Occ. (<1)
O1	0.22429 (7)	−0.0427 (3)	0.23258 (9)	0.0696 (7)	
N1	0.14081 (10)	0.0565 (5)	0.08648 (10)	0.0804 (9)	
C1A	0.12806 (11)	0.1206 (6)	0.01625 (9)	0.0610 (11)	0.869 (3)
C2A	0.16207 (10)	0.3042 (5)	0.00568 (9)	0.0595 (11)	0.869 (3)
C3A	0.15111 (10)	0.3693 (4)	−0.06272 (11)	0.0649 (12)	0.869 (3)
C4A	0.10612 (11)	0.2509 (5)	−0.12053 (8)	0.0717 (13)	0.869 (3)
C5A	0.07211 (10)	0.0673 (6)	−0.10996 (10)	0.0792 (14)	0.869 (3)
C6A	0.08307 (11)	0.0022 (5)	−0.04157 (12)	0.0756 (16)	0.869 (3)
C7A	0.20928 (14)	0.4464 (7)	0.06895 (17)	0.0812 (12)	0.869 (3)
C8A	0.18663 (15)	0.5678 (7)	−0.07597 (18)	0.0871 (16)	0.869 (3)
C9	0.11296 (10)	0.1723 (5)	0.12023 (12)	0.0553 (8)	
C10	0.12760 (10)	0.0941 (5)	0.18928 (12)	0.0528 (8)	
C11	0.09907 (11)	0.2087 (6)	0.22200 (13)	0.0679 (10)	
C12	0.05732 (12)	0.3965 (6)	0.18848 (15)	0.0765 (11)	
C13	0.04389 (11)	0.4728 (6)	0.12102 (14)	0.0737 (11)	
C14	0.07117 (10)	0.3638 (5)	0.08703 (13)	0.0658 (10)	
C15	0.17026 (11)	−0.1194 (5)	0.22527 (13)	0.0637 (10)	
C6B	0.1812 (8)	0.377 (4)	0.0253 (7)	0.0610 (11)	0.131 (3)
C7B	0.0593 (8)	−0.096 (4)	−0.0260 (10)	0.0610 (11)	0.131 (3)
C1B	0.1440 (9)	0.194 (4)	0.0291 (8)	0.0610 (11)	0.131 (3)
C2B	0.1000 (8)	0.091 (4)	−0.0326 (10)	0.0610 (11)	0.131 (3)
C3B	0.0933 (7)	0.172 (4)	−0.0982 (8)	0.0610 (11)	0.131 (3)
C4B	0.1305 (7)	0.355 (3)	−0.1021 (7)	0.0610 (11)	0.131 (3)
C5B	0.1745 (6)	0.457 (3)	−0.0404 (8)	0.0610 (11)	0.131 (3)
C8B	0.0445 (7)	0.065 (4)	−0.1661 (9)	0.0610 (11)	0.131 (3)
H1	0.16684	−0.05878	0.10922	0.0964*	
H1A	0.24482	−0.17371	0.24238	0.0836*	
H8C	0.22583	0.52079	−0.04878	0.1304*	0.869 (3)
H11	0.10832	0.15763	0.26794	0.0813*	
H12	0.03857	0.47020	0.21140	0.0918*	
H13	0.01593	0.59991	0.09808	0.0884*	
H14	0.06171	0.41825	0.04127	0.0790*	
H15A	0.17367	−0.15338	0.27172	0.0764*	
H15B	0.15780	−0.28213	0.19786	0.0764*	
H4A	0.09879	0.29446	−0.16629	0.0860*	0.869 (3)
H5A	0.04201	−0.01196	−0.14864	0.0953*	0.869 (3)
H6A	0.06032	−0.12068	−0.03449	0.0906*	0.869 (3)
H7A	0.24531	0.38724	0.07493	0.1218*	0.869 (3)
H7B	0.20635	0.40668	0.11094	0.1218*	0.869 (3)
H7C	0.20588	0.63470	0.06078	0.1218*	0.869 (3)
H8A	0.18057	0.74134	−0.06220	0.1304*	0.869 (3)
H8B	0.17627	0.56839	−0.12545	0.1304*	0.869 (3)
H4B	0.12604	0.40865	−0.14600	0.0732*	0.131 (3)
H5B	0.19942	0.57989	−0.04293	0.0732*	0.131 (3)
H6B	0.21064	0.44540	0.06659	0.0732*	0.131 (3)
H7D	0.04522	−0.22049	−0.06458	0.0914*	0.131 (3)
H7E	0.02823	0.00255	−0.02709	0.0914*	0.131 (3)
H7F	0.07823	−0.19112	0.01807	0.0914*	0.131 (3)
H8D	0.04325	−0.12510	−0.16349	0.0914*	0.131 (3)
H8E	0.04981	0.11462	−0.20581	0.0914*	0.131 (3)
H8F	0.00953	0.13853	−0.17175	0.0914*	0.131 (3)

### Atomic displacement parameters (Å^2^)

**Table:**

	*U*^11^	*U*^22^	*U*^33^	*U*^12^	*U*^13^	*U*^23^
O1	0.0578 (11)	0.0489 (11)	0.0796 (12)	0.0012 (8)	0.0139 (9)	0.0087 (9)
N1	0.0957 (17)	0.0923 (18)	0.0569 (14)	0.0498 (14)	0.0392 (13)	0.0256 (11)
C1A	0.066 (2)	0.064 (2)	0.0521 (19)	0.0178 (16)	0.0271 (18)	0.0012 (15)
C2A	0.065 (2)	0.059 (2)	0.0446 (17)	0.0215 (16)	0.0177 (15)	−0.0045 (14)
C3A	0.077 (2)	0.064 (2)	0.050 (2)	0.0204 (17)	0.0270 (18)	0.0003 (15)
C4A	0.088 (3)	0.075 (2)	0.0440 (18)	0.0262 (19)	0.0245 (18)	0.0037 (16)
C5A	0.072 (2)	0.092 (3)	0.052 (2)	0.0098 (19)	0.0116 (19)	−0.0105 (18)
C6A	0.070 (3)	0.085 (3)	0.065 (2)	0.0081 (19)	0.0263 (19)	−0.0012 (19)
C7A	0.081 (2)	0.079 (2)	0.072 (2)	0.0014 (18)	0.0267 (19)	−0.0118 (18)
C8A	0.101 (3)	0.084 (3)	0.085 (2)	0.014 (2)	0.051 (2)	0.0154 (19)
C9	0.0571 (14)	0.0540 (16)	0.0499 (14)	0.0065 (12)	0.0212 (12)	0.0020 (11)
C10	0.0545 (14)	0.0471 (15)	0.0481 (14)	−0.0076 (11)	0.0171 (12)	−0.0010 (11)
C11	0.0721 (18)	0.078 (2)	0.0509 (15)	−0.0144 (15)	0.0268 (14)	−0.0061 (13)
C12	0.0680 (19)	0.091 (2)	0.073 (2)	0.0069 (16)	0.0353 (16)	−0.0131 (16)
C13	0.0614 (17)	0.082 (2)	0.0659 (18)	0.0188 (14)	0.0204 (14)	−0.0078 (14)
C14	0.0661 (17)	0.0721 (19)	0.0509 (15)	0.0180 (14)	0.0208 (13)	0.0061 (12)
C15	0.0654 (17)	0.0543 (17)	0.0579 (16)	−0.0070 (13)	0.0181 (13)	0.0070 (12)
C6B	0.066 (2)	0.064 (2)	0.0521 (19)	0.0178 (16)	0.0271 (18)	0.0012 (15)
C7B	0.066 (2)	0.064 (2)	0.0521 (19)	0.0178 (16)	0.0271 (18)	0.0012 (15)
C1B	0.066 (2)	0.064 (2)	0.0521 (19)	0.0178 (16)	0.0271 (18)	0.0012 (15)
C2B	0.066 (2)	0.064 (2)	0.0521 (19)	0.0178 (16)	0.0271 (18)	0.0012 (15)
C3B	0.066 (2)	0.064 (2)	0.0521 (19)	0.0178 (16)	0.0271 (18)	0.0012 (15)
C4B	0.066 (2)	0.064 (2)	0.0521 (19)	0.0178 (16)	0.0271 (18)	0.0012 (15)
C5B	0.066 (2)	0.064 (2)	0.0521 (19)	0.0178 (16)	0.0271 (18)	0.0012 (15)
C8B	0.066 (2)	0.064 (2)	0.0521 (19)	0.0178 (16)	0.0271 (18)	0.0012 (15)

### Geometric parameters (Å, °)

**Table:**

O1—C15	1.435 (4)	C12—C13	1.368 (4)
O1—H1A	0.8200	C13—C14	1.367 (4)
N1—C1A	1.413 (3)	C4A—H4A	0.9300
N1—C1B	1.448 (19)	C4B—H4B	0.9300
N1—C9	1.389 (4)	C5A—H5A	0.9300
N1—H1	0.8600	C5B—H5B	0.9300
C1A—C2A	1.390 (4)	C6A—H6A	0.9300
C1A—C6A	1.390 (3)	C6B—H6B	0.9300
C1B—C6B	1.39 (3)	C7A—H7B	0.9600
C1B—C2B	1.39 (3)	C7A—H7A	0.9600
C2A—C3A	1.390 (3)	C7A—H7C	0.9600
C2A—C7A	1.526 (4)	C7B—H7F	0.9600
C2B—C7B	1.50 (3)	C7B—H7D	0.9600
C2B—C3B	1.39 (3)	C7B—H7E	0.9600
C3A—C8A	1.497 (5)	C8A—H8B	0.9600
C3A—C4A	1.390 (3)	C8A—H8A	0.9600
C3B—C8B	1.52 (2)	C8A—H8C	0.9600
C3B—C4B	1.39 (3)	C8B—H8D	0.9600
C4A—C5A	1.390 (4)	C8B—H8F	0.9600
C4B—C5B	1.39 (2)	C8B—H8E	0.9600
C5A—C6A	1.390 (3)	C11—H11	0.9300
C5B—C6B	1.39 (2)	C12—H12	0.9300
C9—C14	1.392 (4)	C13—H13	0.9300
C9—C10	1.395 (3)	C14—H14	0.9300
C10—C15	1.491 (4)	C15—H15B	0.9700
C10—C11	1.384 (4)	C15—H15A	0.9700
C11—C12	1.379 (4)		
			
O1···N1	2.904 (3)	H1A···H1A^i^	2.5400
O1···C15^i^	3.307 (3)	H1A···C15^ii^	3.0300
O1···O1^ii^	2.796 (2)	H4A···H8B	2.2900
O1···O1^i^	2.796 (2)	H4B···H8E	2.3500
O1···H1	2.3500	H5A···C12^iv^	3.0700
O1···H1A^i^	2.0000	H6A···C13^iv^	3.0800
N1···O1	2.904 (3)	H7A···C8A	2.9900
N1···H7B	2.3700	H7A···H8C^viii^	2.3500
N1···H15B	2.7900	H7A···C8A^viii^	2.9200
N1···H7E	2.8600	H7A···H8C	2.5400
N1···H7F	2.0500	H7B···N1	2.3700
C2B···C14	3.30 (2)	H7B···H1	2.5600
C6A···C14	3.439 (4)	H7B···C9	2.8600
C7A···C9	3.530 (5)	H7C···C1A^vi^	3.0600
C7B···C9	3.073 (19)	H7C···C8A	2.7400
C7B···C14^iii^	3.55 (2)	H7C···H1^vi^	2.3600
C7B···C14	3.25 (2)	H7C···H8A	2.4500
C7B···C13^iv^	3.17 (2)	H7D···H13^iv^	2.4000
C7B···C14^iv^	3.38 (2)	H7D···C14^iv^	3.0100
C8B···C12^iv^	3.44 (2)	H7D···H8D	2.1500
C8B···C8B^v^	3.24 (2)	H7D···C12^iv^	2.9100
C9···C7B	3.073 (19)	H7D···C8B	2.6000
C9···C7A	3.530 (5)	H7D···C13^iv^	2.4600
C12···C8B^iv^	3.44 (2)	H7E···C14	2.8200
C13···C7B^iv^	3.17 (2)	H7E···H14	2.4700
C14···C7B	3.25 (2)	H7E···C9	3.0100
C14···C7B^iv^	3.38 (2)	H7E···N1	2.8600
C14···C7B^vi^	3.55 (2)	H7E···C14^iv^	2.9900
C14···C6A	3.439 (4)	H7E···H13^vii^	2.4500
C14···C2B	3.30 (2)	H7E···C7B^iv^	3.0900
C15···O1^ii^	3.307 (3)	H7E···H7E^iv^	2.3000
C1A···H7C^iii^	3.0600	H7F···C9	2.6600
C1A···H14	2.5700	H7F···C14^iii^	2.7400
C1B···H14	2.6000	H7F···N1	2.0500
C2B···H14	2.7900	H7F···H1	2.3500
C5A···H13^vii^	3.0000	H7F···H14^iii^	2.1200
C5A···H8A^iii^	3.0600	H8A···H7C	2.4500
C6A···H13^vii^	3.0800	H8A···C5A^vi^	3.0600
C6A···H14	2.9700	H8A···C7A	2.9400
C7A···H8C	2.7800	H8B···H4A	2.2900
C7A···H1	3.0700	H8B···H11^xi^	2.5500
C7A···H8C^viii^	3.0700	H8C···C7A	2.7800
C7A···H8A	2.9400	H8C···H7A	2.5400
C7A···H1^vi^	3.0300	H8C···C7A^viii^	3.0700
C7B···H1	2.9700	H8C···H7A^viii^	2.3500
C7B···H8D	2.7700	H8D···C12^iv^	2.8400
C7B···H7E^iv^	3.0900	H8D···C7B	2.7700
C7B···H14^iii^	2.8200	H8D···H7D	2.1500
C7B···H14	2.9500	H8D···H12^iv^	2.6000
C7B···H8F	3.0000	H8E···H8E^v^	2.4400
C8A···H7A^viii^	2.9200	H8E···H4B	2.3500
C8A···H7C	2.7400	H8E···C8B^v^	2.7300
C8A···H7A	2.9900	H8E···C11^ix^	2.9500
C8B···H8F^v^	3.0900	H8E···H11^ix^	2.3400
C8B···H11^ix^	2.9100	H8E···H8F^v^	2.3400
C8B···H12^vii^	3.0500	H8F···C12^vii^	2.8700
C8B···H8E^v^	2.7300	H8F···C13^vii^	2.9200
C8B···H7D	2.6000	H8F···C7B	3.0000
C9···H7B	2.8600	H8F···C8B^v^	3.0900
C9···H7E	3.0100	H8F···H8E^v^	2.3400
C9···H7F	2.6600	H8F···H12^vii^	2.2900
C11···H8E^x^	2.9500	H8F···H13^vii^	2.3900
C12···H5A^iv^	3.0700	H11···H15A	2.3200
C12···H8D^iv^	2.8400	H11···H8E^x^	2.3400
C12···H15B^vi^	3.0700	H11···H8B^xii^	2.5500
C12···H7D^iv^	2.9100	H11···C8B^x^	2.9100
C12···H8F^vii^	2.8700	H12···H8D^iv^	2.6000
C13···H6A^iv^	3.0800	H12···C8B^vii^	3.0500
C13···H8F^vii^	2.9200	H12···H8F^vii^	2.2900
C13···H15B^vi^	2.9700	H13···C5A^vii^	3.0000
C13···H7D^iv^	2.4600	H13···C6A^vii^	3.0800
C14···H15B^vi^	3.0000	H13···H8F^vii^	2.3900
C14···H7E	2.8200	H13···H7D^iv^	2.4000
C14···H7F^vi^	2.7400	H13···H7E^vii^	2.4500
C14···H7D^iv^	3.0100	H14···C1A	2.5700
C14···H7E^iv^	2.9900	H14···C2B	2.7900
C15···H1	2.4600	H14···C6A	2.9700
C15···H1A^i^	3.0300	H14···C1B	2.6000
H1···C15	2.4600	H14···H7F^vi^	2.1200
H1···H7C^iii^	2.3600	H14···H7E	2.4700
H1···H15B	2.3200	H14···C7B	2.9500
H1···H7B	2.5600	H14···C7B^vi^	2.8200
H1···H7F	2.3500	H15A···H11	2.3200
H1···C7A^iii^	3.0300	H15B···C13^iii^	2.9700
H1···C7A	3.0700	H15B···C14^iii^	3.0000
H1···C7B	2.9700	H15B···H1	2.3200
H1···O1	2.3500	H15B···C12^iii^	3.0700
H1A···O1^ii^	2.0000	H15B···N1	2.7900
H1A···H1A^ii^	2.5400		
			
C15—O1—H1A	109.00	C4A—C5A—H5A	120.00
C1B—N1—C9	120.2 (9)	C6A—C5A—H5A	120.00
C1A—N1—C9	122.6 (2)	C4B—C5B—H5B	120.00
C1B—N1—H1	116.00	C6B—C5B—H5B	120.00
C1A—N1—H1	119.00	C5A—C6A—H6A	120.00
C9—N1—H1	119.00	C1A—C6A—H6A	120.00
N1—C1A—C6A	121.5 (3)	C1B—C6B—H6B	120.00
C2A—C1A—C6A	120.00 (19)	C5B—C6B—H6B	120.00
N1—C1A—C2A	118.5 (2)	C2A—C7A—H7C	109.00
C2B—C1B—C6B	120.2 (16)	C2A—C7A—H7B	109.00
N1—C1B—C2B	105.3 (17)	C2A—C7A—H7A	109.00
N1—C1B—C6B	134.4 (14)	H7B—C7A—H7C	109.00
C1A—C2A—C3A	120.0 (2)	H7A—C7A—H7B	109.00
C3A—C2A—C7A	119.9 (2)	H7A—C7A—H7C	109.00
C1A—C2A—C7A	120.0 (2)	C2B—C7B—H7D	110.00
C1B—C2B—C7B	118.3 (18)	H7E—C7B—H7F	109.00
C1B—C2B—C3B	120 (2)	C2B—C7B—H7E	110.00
C3B—C2B—C7B	121.8 (18)	C2B—C7B—H7F	110.00
C2A—C3A—C4A	120.0 (2)	H7D—C7B—H7E	109.00
C4A—C3A—C8A	118.7 (2)	H7D—C7B—H7F	109.00
C2A—C3A—C8A	121.4 (2)	C3A—C8A—H8C	109.00
C2B—C3B—C4B	120.1 (16)	H8A—C8A—H8B	109.00
C2B—C3B—C8B	120.2 (18)	C3A—C8A—H8B	109.00
C4B—C3B—C8B	119.7 (14)	C3A—C8A—H8A	109.00
C3A—C4A—C5A	120.01 (18)	H8A—C8A—H8C	109.00
C3B—C4B—C5B	120.0 (14)	H8B—C8A—H8C	109.00
C4A—C5A—C6A	120.0 (2)	C3B—C8B—H8F	109.00
C4B—C5B—C6B	120.1 (16)	C3B—C8B—H8D	110.00
C1A—C6A—C5A	120.0 (3)	C3B—C8B—H8E	110.00
C1B—C6B—C5B	119.9 (15)	H8E—C8B—H8F	109.00
N1—C9—C10	118.8 (2)	H8D—C8B—H8F	109.00
C10—C9—C14	119.5 (2)	H8D—C8B—H8E	110.00
N1—C9—C14	121.7 (2)	C10—C11—H11	119.00
C9—C10—C11	118.2 (2)	C12—C11—H11	119.00
C11—C10—C15	120.7 (2)	C11—C12—H12	120.00
C9—C10—C15	120.9 (2)	C13—C12—H12	120.00
C10—C11—C12	121.9 (2)	C12—C13—H13	120.00
C11—C12—C13	119.1 (3)	C14—C13—H13	120.00
C12—C13—C14	120.6 (3)	C9—C14—H14	120.00
C9—C14—C13	120.7 (2)	C13—C14—H14	120.00
O1—C15—C10	110.6 (2)	O1—C15—H15B	110.00
C3A—C4A—H4A	120.00	H15A—C15—H15B	108.00
C5A—C4A—H4A	120.00	C10—C15—H15A	110.00
C5B—C4B—H4B	120.00	C10—C15—H15B	110.00
C3B—C4B—H4B	120.00	O1—C15—H15A	110.00
			
C9—N1—C1A—C2A	98.1 (3)	C3A—C4A—C5A—C6A	0.0 (4)
C9—N1—C1A—C6A	−82.7 (4)	C4A—C5A—C6A—C1A	0.0 (4)
C1A—N1—C9—C10	179.0 (3)	N1—C9—C10—C11	−179.3 (3)
C1A—N1—C9—C14	−1.2 (4)	N1—C9—C10—C15	−3.0 (4)
N1—C1A—C2A—C3A	179.2 (3)	C14—C9—C10—C11	0.9 (4)
N1—C1A—C2A—C7A	−4.4 (4)	C14—C9—C10—C15	177.2 (2)
C6A—C1A—C2A—C3A	0.0 (4)	N1—C9—C14—C13	179.3 (3)
C6A—C1A—C2A—C7A	176.4 (3)	C10—C9—C14—C13	−0.9 (4)
N1—C1A—C6A—C5A	−179.2 (3)	C9—C10—C11—C12	−0.3 (4)
C2A—C1A—C6A—C5A	0.0 (4)	C15—C10—C11—C12	−176.5 (3)
C1A—C2A—C3A—C4A	0.0 (4)	C9—C10—C15—O1	62.0 (3)
C1A—C2A—C3A—C8A	179.5 (3)	C11—C10—C15—O1	−121.8 (3)
C7A—C2A—C3A—C4A	−176.4 (3)	C10—C11—C12—C13	−0.3 (5)
C7A—C2A—C3A—C8A	3.1 (4)	C11—C12—C13—C14	0.4 (5)
C2A—C3A—C4A—C5A	0.0 (4)	C12—C13—C14—C9	0.3 (4)
C8A—C3A—C4A—C5A	−179.5 (3)		

Symmetry codes: (i) −*x*+1/2, *y*+1/2, −*z*+1/2; (ii) −*x*+1/2, *y*−1/2, −*z*+1/2; (iii) *x*, *y*−1, *z*; (iv) −*x*, −*y*, −*z*; (v) −*x*, *y*, −*z*−1/2; (vi) *x*, *y*+1, *z*; (vii) −*x*, −*y*+1, −*z*; (viii) −*x*+1/2, −*y*+1/2, −*z*; (ix) *x*, −*y*, *z*−1/2; (x) *x*, −*y*, *z*+1/2; (xi) *x*, −*y*+1, *z*−1/2; (xii) *x*, −*y*+1, *z*+1/2.

### Hydrogen-bond geometry (Å, °)

**Table:**

Cg1 and Cg2 are the centroids of the C1A—C6A and C9—C14 rings, respectively.

**Table:**

*D*—H···*A*	*D*—H	H···*A*	*D*···*A*	*D*—H···*A*
N1—H1···O1	0.86	2.35	2.904 (3)	123
O1—H1A···O1^ii^	0.82	2.00	2.796 (2)	163
C8A—H8A···Cg1^vi^	0.96	2.88	3.783 (4)	157
C15—H15B···Cg2^iii^	0.97	2.77	3.634 (3)	148

Symmetry codes: (ii) −*x*+1/2, *y*−1/2, −*z*+1/2; (vi) *x*, *y*+1, *z*; (iii) *x*, *y*−1, *z*.
